# The Impact of Isoinertial Training on Thigh Muscle Volume: Ultrasound and Dynamometric Evaluation

**DOI:** 10.3390/bioengineering12121321

**Published:** 2025-12-04

**Authors:** Ligia Rusu, Aurora Dobre Ungureanu, Alexandru Chivaran, Mihnea Ion Marin, Mihai Robert Rusu, Andrei Spinu, Mara Marin, Gabriel Buciu, Mirela Lucia Calina

**Affiliations:** 1 Department of Sport Medicine and Physical Therapy, University of Craiova, 200585 Craiova, Romania; chivaranalexandru94@gmail.com (A.C.); mirela.calina@edu.ucv.ro (M.L.C.); 2 Department of Theory and Motric Activity, University of Craiova, 200585 Craiova, Romania; aurora.ungureanu@edu.ucv.ro (A.D.U.); mihai.rusu@edu.ucv.ro (M.R.R.); 3 Faculty of Mechanics, University of Craiova, 200585 Craiova, Romania; mihnea.marin@edu.ucv.ro; 4 Faculty of Medicine, University of Medicine and Pharmacy, 200349 Craiova, Romania; spinu.andreeei@yahoo.com; 5 Department of Informatics, S.C.Poem Metaphore S.R.L., 032346 Bucharest, Romania; marra.marin@gmail.com; 6 Department of General Nursing, Faculty of Nursing-Târgu Jiu, University Titu Maiorescu, 021251 Bucharest, Romania; gabriel.buciu@prof.utm.ro

**Keywords:** muscle strength, isoinertial, power output, neural adaptation

## Abstract

Muscle imbalance due to reduced muscular endurance is a significant risk factor. Thus, for the lower limb, muscle imbalance is one of the most common causes of traumatic injury. A number of studies on isoinertial technology have demonstrated that it allows the development of forces similar to or greater than those generated in the same exercise but performed with traditional weights. Our research aimed to analyze the morpho-functional changes at the muscle level using ultrasound, and the evolution of muscle power output express of maximal muscle strength at the level of the knee extensors, specifically the rectus femoris muscle, following an isoinertial training program. The study included 11 female soccer-practicing sportswomen with average age (15.18 ± 1.08). The assessment included an ultrasound assessment of the rectus femoris muscle, before and after isoiniertial training (post acute moment); a muscle force assessment using dynamometry; and an evaluation of isoinertial parameters as concentric and excentric power, in terms of coefficients that represent report between the left and right sides. The isoinertial protocols training included three weekly sessions of isoinertial exercises. The results show an increase in the values of the ultrasound dimensions, approximately at the same level for both measured dimensions, which is significant for demonstrating the existence of an increase in muscle volume. An important progress is observed in the mean maximum strength and maximum force, but especially in the duration of maintenance of the maximum loading force. There is no statistically significant symmetry at the level of the rectus femoris muscle for concentric power and no statistically significant symmetry tendency for eccentric power, although there is a favorable evolution in terms of values.

## 1. Introduction

Compared to other team sports, football has the highest risk of injury, with 70% of injuries being muscle injuries and localized to the lower extremities [1]. Ekstrand et al. [2] stated that 92% of muscle injuries affect the four major muscle groups of the lower limbs: hamstrings (37%), adductors (23%), quadriceps (19%) and calf muscles (13%). A total of 16% of reported muscle injuries are repetitive injuries, which impose restrictions in sports activity due to the long recovery period.

Muscle imbalance due to reduced muscular endurance is a significant risk factor. Thus, in the lower limb, muscle imbalance between agonists and antagonists, i.e., between hamstrings and quadriceps and/or a lack of hamstring strength, is one of the most common causes of traumatic injury [3,4,5].

According to Tais [6], eccentric training leads to changes in muscle structure and is considered useful for improving eccentric muscle strength. It has been shown that eccentric exercises can reduce the injury rate because muscle injuries occur when the length of muscle fibers exceeds the optimal length; therefore, injuries can be reduced, where the optimal length can be increased. Clark [7] demonstrated in one of his studies that this length consistently increases with eccentric exercise.

Isoinertial technology is one of the most recent ways of increasing eccentric loading, which can also be considered an innovative form of strength training. It is based on the use of rotating systems to provide inertial resistance, independent of gravity [8].

A number of studies on isoinertial technology have demonstrated that it allows the development of forces similar to or greater than those generated in the same exercise but performed with traditional weights [9,10].

In terms of the benefits of using isoinertial technology in the treatment or management of certain pathologies, there are studies that demonstrate its effectiveness in recovery training after anterior cruciate ligament tears [11], patellar tendinopathy [12], and after muscle and tendon injuries caused by muscle imbalances. Regarding muscle behavior under the dynamic loading, many studies speak about the viscoelastic intrinsic models that explain what is happening during dynamic loading and what the material response could be. In this way authors such as Datao et al. [13], have tried to create a deep learning model that integrates coupled kinematics and dynamics. In this way, the authors are using the real-time inversion of ligament and muscle loading force failure states.

Similarly, isoinertial technology has also proven its effectiveness in the process of bone formation and slowing the loss of bone mass, as well as in increasing the cross-sectional area of the muscle during periods of prolonged immobilization, which is important for the mechanical stimulus generated [14]. Regarding isoinertial training, there are studies that present the results of different approaches of isoinertial training, such as the study by Piqueras-Sanchiz et al. [15] which tested both men and women during four test sessions of the isoinertial exercise of calf flexion exercise on the thigh using different inertial loads (0.083, 0.132, 0.182, 0.266 and 0.350 kg/m^2^). It was concluded that in order to obtain reliable and stable results, a familiarization process with the isoinertial exercise of calf flexion exercise on the thigh with inexperienced subjects is necessary, recommending two to four sessions depending on experience.

The isoinertial training method that can be used to improve muscle hypertrophy [16], neuromuscular function [17], muscle strength [18], and sprint time [19] owes its effectiveness to tailored resistance and individualized, optimal eccentric overload [20]. The intervention is based on the resistance generated by an isoinertial equipment that applies eccentric overloads during the athlete’s movements.

Although the acceptability and implicitly of the use of this type of muscle training has increased, there is little scientific evidence to draw conclusions on the recommendations of this type of training, whose effects must be estimated in two aspects: the immediate effect (acute post-strength phase) and the effect of prolonged training.

To the same extent, there are few studies that objectivize the morpho-functional changes that can occur after isoinertial training, and especially the acute response to isoinertial training. One of the ways of assessing muscle behavior in response to isoinertial training is musculoskeletal ultrasonography combined with dynamometry. Starting from this aspect in the literature, the quantification of muscle function using ultrasonography (USMK) has been questioned and an increase in muscle thickness and a decrease in muscle length have been observed. Monitoring the change in muscle thickness during contractions would be a useful tool to assess muscle contractile properties [21]. Several studies have provided evidence that relative changes in muscle contractile parameters have been associated with skeletal muscle atrophy [22], percentage of muscle fiber types, and general and local muscle fatigue [21]. The work of Guo et al. [23] may be one of the earliest that used single-element ultrasonography to track changes of thickness during skeletal muscle contraction. They tracked the extensor carpi radialis extensor muscle of the carpus that controls motion in the fist.

These ultrasound features may represent a way to quantify muscle function after isoinertial training.

According to the literature after a session of isoinertial training, a post-activation potentiation enhancement (PAPE) effects are developed, which refers to an acute enhancement of athletic performance following a pre-load activity, based on well-described phenomenon with a short half-life (~28 s) that enhances muscle force production at submaximal levels of calcium saturation [24]. According to the study of de Lacalle et al., [24] the results revealed significant differences (*p* < 0.01) in the ultrasound changes in the supraspinatus tendon cross-sectional area (CSA), measured by ultrasound, and in tendon volume after 10 repetitions of maximal force. During isoinertial training, the extravasation of water could be based much more on the straightening of the curl as well as the realignment and stretching of collagen. This is followed by the production of lateral compressive forces between the fibrils and a reduction in the interfibrillar space, resulting in positive hydrostatic pressure and, therefore, the movement of fluids out [25].

Authors such as Annibalini et al. [26] demonstrated that the FWT exercise increases muscular and systemic inflammatory responses and muscle remodeling marker levels. The main finding of their descriptive study is that FWT exercise acutely affects the local and systemic markers involved in late structural remodeling and functional adaptation of skeletal muscle. Also, cell communication is affected by FWT due to the cytokines liberated during the FWT, and mediate gene expression in the target cells [27]. One of these is the miR-206, along with other “myomiRNAs” such as miR-1 and miR-133b, which is specific to or enriched in the skeletal muscle tissue and has roles in the regulation of muscle development and differentiation [28]. Therefore, FWT based isoinertial exercise seems to induce early muscle and systemic molecular adaptations, even in trained individuals, which have the potential to induce a late hypertrophic response.

Hu et al. [29] made a metanalysis of a comparative study about the effects of isoinertial flywheel training (FWT) and traditional resistance training, discussing the morphological and neural-adaptive changes in the human body. Morphological changes included increases in power output, increase in muscle cross-sectional area, musculotendinous stiffness, and also an increase and improvement of motor unit recruitment and neuromuscular inhibition [30].

Regarding isoinertial training, there is a lack of research about how to provide precise recommendations on how to accurately design and prescribe flywheel exercises using a systematic approach especially in elite sport athletes, as Beato et al. highlighted in their paper [31].

There is much more research in the field of sport activity regarding how to recommend FWT; however, this is still less than in clinical assessment. One study is the research of Ćorilić et al. [32], which investigated the results and effects of six weeks of isoinertial training on male tennis players and observed an improvement in the ability of tennis players when it came to linear speed.

Muscle imbalance is also a challenge in FWT especially in the rehabilitation process, as Patra et al. observed in their study about isoinertial training in post ACL reconstruction. They found that four weeks of isoinertial training, 30 min twice a week, produced a resemblance to dynamic loading and functional movement patterns may lead to greater enhancements in isometric strength and balance [33]. 

Our research aimed to analyze the morpho-functional changes at the muscle level using ultrasonography, and the evolution of muscle strength at the level of the knee extensors, specifically the rectus femoris muscle, following an isoinertial training program.

## 2. Materials and Methods

### 2.1. Subjects

The study included 11 female soccer-practicing sportswomen average ± SD: age (15.18 ± 1.08), height (162 ± 6.64), weight (53.45 ± 8.13), BMI (21.34 ± 3.61). The sportswomen had about 4 years of experience as members of the female soccer players of League 1 at the University of Craiova Sports Club; the research was carried out in the INCESA Laboratory of Innovative Techniques and Pieces (www.incesa.ro, Craiova, Romania). Respecting the rules of research ethics, for each of the subjects an informed consent was signed by their parents. Both subjects and parents were made aware of the examination protocol, that the assessments are non-invasive, and that personal data comply with GDPR rules. In addition, the principles of the Declaration of Helsinki on Human Subjects Research (v 2013) were followed. The study received approval from the local Research Ethics 154 and Deontology Commission of the University of Craiova (approval no 579/2025).

Selections were made based on the following criteria:

*Inclusion criteria:*

- Playing soccer for at least 1 year;
- Constant participation in training;
- Willingness to be included in an isoinertial training program.

*Exclusion Criteria:*

- Injured within the last year;
- Inability to participate in testing;
- Also participate in other sports.

### 2.2. Evaluation

#### 2.2.1. Ultrasound Assessment

**Figure 1:**
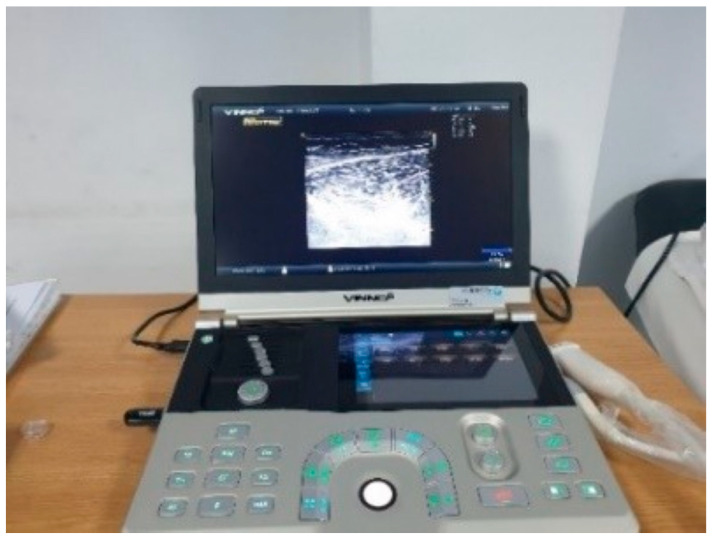
Ultrasound machine VINNO 6.

The ultrasound scans were performed using VINNO 6 Color Doppler VINNO 6 equipment, linear probe X4-12L, 8–16 Hz, Gain: 45 dB, Dynamic Range: 72, mode B (Figure 1), and the examination was carried out at the level of the rectus femoris muscle, bilaterally, in the area of maximum muscle relief. The athletes were in **supine** with a slight hip flexion. We used a pillow under the knee for comfort and better visualization.

This position relaxed the quadriceps and facilitated the scanning of the rectus femoris. After cleaning the skin surface, we used Aquasonic 100 ultrasound transmission gel. We began with **transverse (short-axis)** scans to assess muscle thickness and echotexture and switched to **longitudinal (long-axis)** scans for tendon continuity and attachment visualization. We used dynamic maneuvers (gentle knee flexion/extension) to confirm tendon integrity. We also performed a post-cut ultrasound evaluation, more specifically before and after an isoinertial training session, to analyze how the muscle group responded immediately to the post-load.

For the rectus femoris muscle, the area of examination corresponded to the mid-distance between the anterosuperior iliac spine and the superior border of the patella.

**Figure 2:**
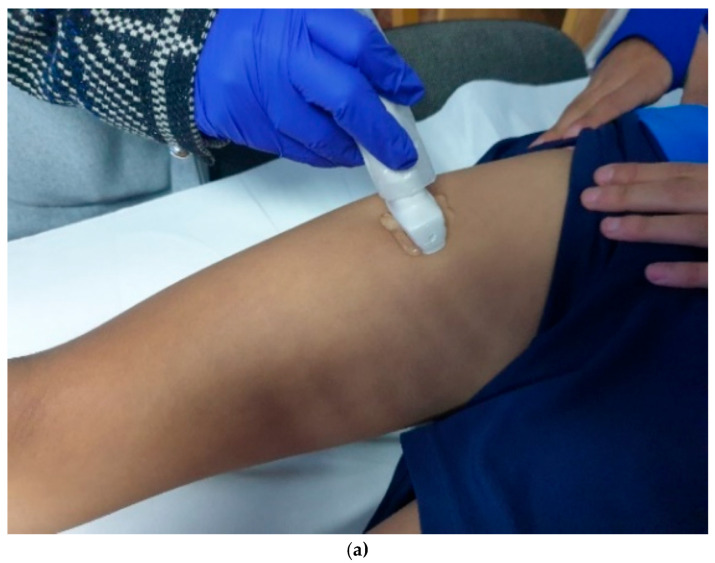

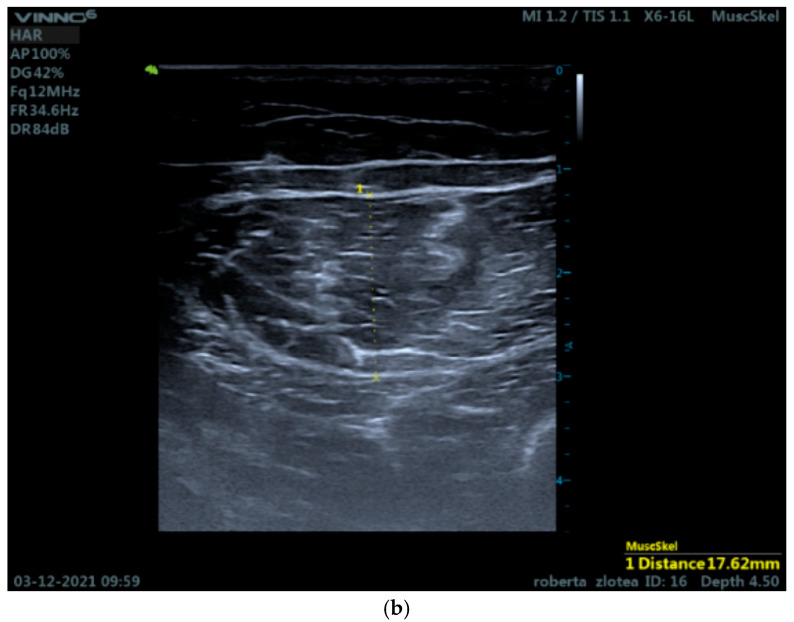
(**a**). The examination of rectus femoris muscle. (**b**). Transversal section examination rectus femoris muscle.

**Figure 3:**
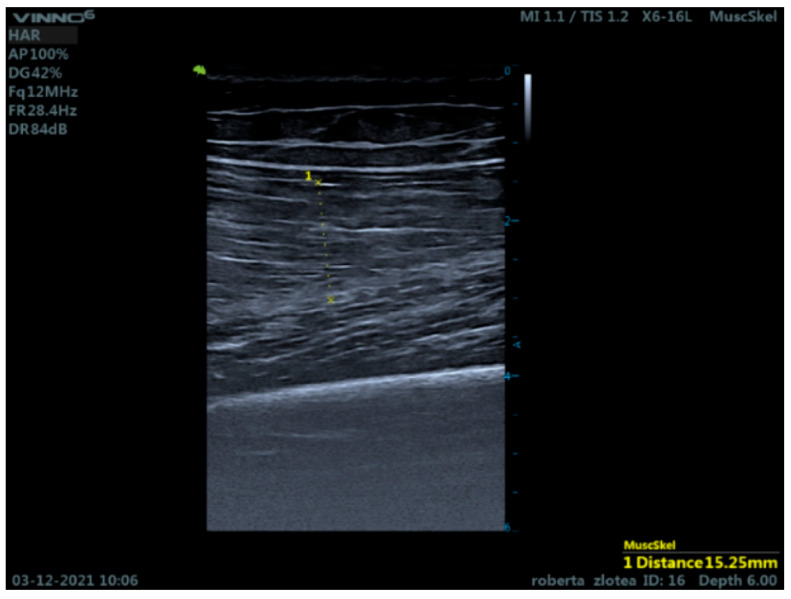
Longitudinal section examination rectus femoris muscle.

Ultrasound evaluation was performed in cross-sectional and longitudinal sections for the rectus femoris (Figure 2b and Figure 3) before and immediately after isoinertial training. Three measurements were recorded for each area that was examined and then they were averaged.

#### 2.2.2. Assessment of Muscle Strength by Dynamometry Used the BioFET Dynamometer V3 with Bluetooth 4.0 Connection

**Figure 4:**
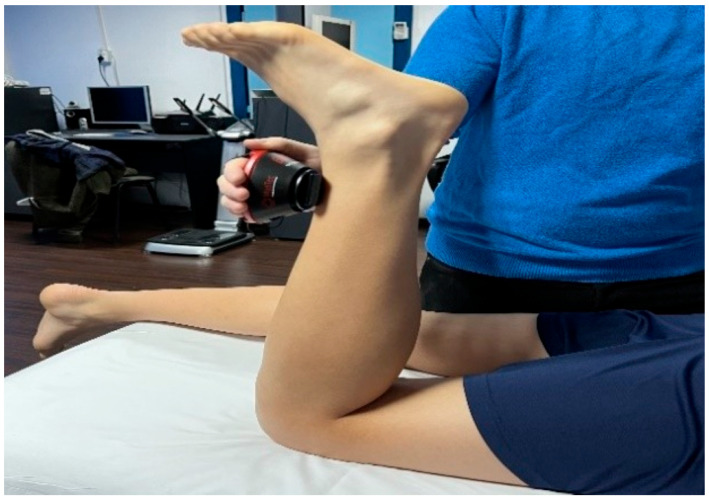
Rectus femoris muscle testing.

The assessment protocol consisted of:

- A test phase for the subject to understand what was going to be performed;
- The test was performed with the subject in the prone position, to test the rectus femoris muscle (Figure 4), placing the dynamometer surface at the level of the lower third of the calf.

#### 2.2.3. Isoinertial Parameters Assessment

The isoinertial system gives the possibility to assess the power output in both phase **concentric and eccentric force, which corresponds to peak** force during the **push phase** (concentric) and **braking phase** (eccentric). The tests performed, which allowed us to assess the specific parameters, were familiarization tests with the Desmotec device which had the *r*ol to help the subject get used to the device (*duration*: 3 series with 15 repetitions each, *rest*: 30 s after each series.). *Maximum force evaluation test—*a very quick test that allows the evaluation of the maximum force expressed during the exercise with a defined inertial load (1 set, 10 repetitions). At the end of the test, the screen displayed the maximum concentric and eccentric force.

Based on this, we calculated some coefficients, as follows:

***Symmetry coefficient concentric power/eccentric power***

The coefficient R1 is the ratio of the mean value of concentric power to the mean value of eccentric power. R1 assesses balance in strength and power output between these two phases.

Coefficient R1 is presented for each muscle group (right quadriceps, left quadriceps), before and after the training program, for the group of 11 athletes.

***Left/right symmetry coefficient of concentric and eccentric power***

Coefficient R2 and R3 are left/right symmetry coefficients and represent the ratio between the mean value of the concentric and eccentric power, respectively, determined on the left lower limb, compared to the mean value of the concentric and eccentric power, respectively, determined on the right lower limb, for the same muscle group—Quadriceps (Qu).

R2 and R3 represent the ratio between the mean value of the concentric and eccentric power left and right side. R2 and R3 ratio may indicate poor control during deceleration and we observe that a small asymmetry exists between left and right side.

### 2.3. Isoinertial Training

**Figure 5:**
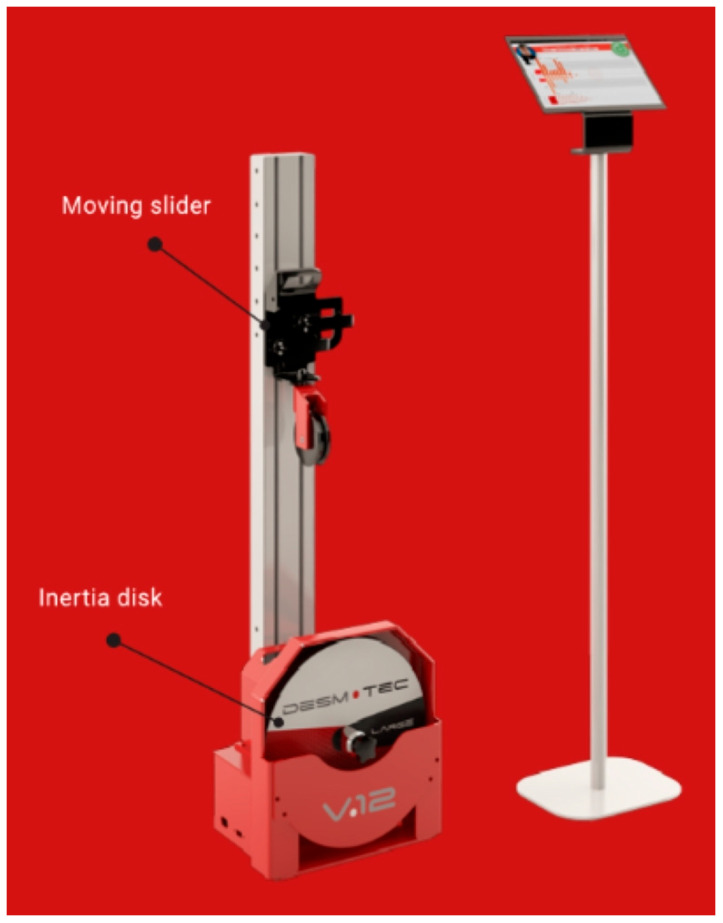
System Desmotec V.Sport (Desmotec: Tecnologia Isoinerziale per Fisioterapia, Sport e Fitness.

Isoinertial training was performed using the *Desmotec V. Sport* equipment, a portable vertical device equipped with disks of different sizes, as well as the dedicated and tablet (Figure 5) to track and store the training sessions in the Cloud. Large disks (large, pro) were used to increase muscle strength and endurance.

The training program included 3 weekly sessions of isoinertial exercises for 8 weeks.

Each athlete performed 3 sets of 10 repetitions each with a 20 s pause between sets for each of the two lower limbs, with the aim of developing quadriceps muscle strength and endurance. The athletes adopted a seated position on a bench; the ankle strap was attached to the distal part of the calf. Once the ankle strap was fixed, it allowed the athletes to perform knee extension against the resistance produced by the device. The Pro disk was used.

The average training session lasted approximately 15 min per athlete.

**Figure 6:**
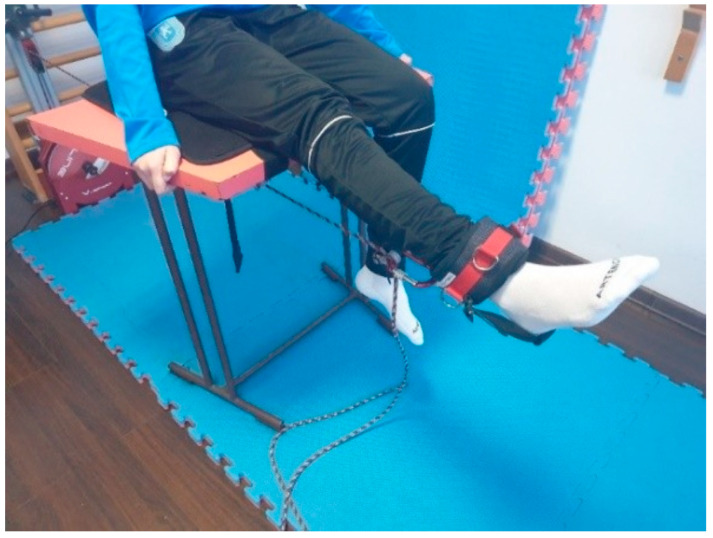
Knee extension using isoinertial system.

The isoinertial training program, as previously mentioned included two categories of exercises aimed at developing eccentric muscle strength and muscle control. The initiation of the isoinertial training program was preceded by the evaluation of the morpho-functional behavior of the trained muscle group as an acute response to the isoinertial training shown in Figure 6, which demonstrates knee extension from the sitting position.

**Execution technique**: female athlete in sitting position, performing knee extension. The ankle is fixed at the distal part of the calf. Attached to the ankle is a strap that connects to the Desmotec device.

Resistance exercise was performed on a seated knee extension flywheel ergometer (Berg and Tesch, 1994) [34]. Each supervised session consisted of four sets of seven maximal, coupled concentric extensions, and eccentric flexions of the knee from about 90 degree to 160–170 degree knee joint angle.

### 2.4. Statistical Analysis

The statistical analysis included descriptive statistics and a Student’s *t*-test. The Student’s *t*-test was applied to reveal any differences between parameter values from evaluation moment 1 (T1) to evaluation moment 2 (T2). The test indicated whether there was a significant difference. We applied the Student’s *t*-test [35] for equal means [36]. To analyze the effect size of the parameter evolution, we used the Cohen D coefficient [37], using the same software [36].

### 2.5. Sample Size Justification

Although the sample size (n = 11) may appear limited, it is justified by several factors. First, this is a proof-of-concept study evaluating novel isoinertial technology with limited prior research, where the primary objective is to demonstrate the existence of an effect rather than precise population parameter estimation. Second, the study population is highly homogeneous (elite female soccer players from League 1, with similar training status and age: 15.18 ± 1.08 years), resulting in reduced within-group variability (coefficient of variation 11–21% for most measurements). Third, post hoc power analysis demonstrated that the observed large effect sizes (Cohen’s d ranging from 0.80 to 1.48) compensated for the smaller sample size, yielding statistical power between 0.86 and 0.99 for all significant tests (calculated using G*Power 3.1), well above the recommended threshold of 0.80, Cohen, 1988 [37]. Similar sample sizes (n = 8–15) have been reported in comparable studies of isoinertial training technology, Naczk et al. [38]. The combination of large effect sizes, high statistical power, low *p*-values (most *p* < 0.001), and consistent results across multiple measurements supports the robustness of our findings despite the limited sample size.

## 3. Results

### 3.1. Results of Ultrasound Recordings

#### Descriptive Analysis of the Ultrasound Recordings

As mentioned above, the ultrasound measurements of the muscle group were performed before isoinertial training and immediately after it. The recorded values are presented in a descriptive statistic, for longitudinal and cross-section, in Table 1.

**Figure 7:**
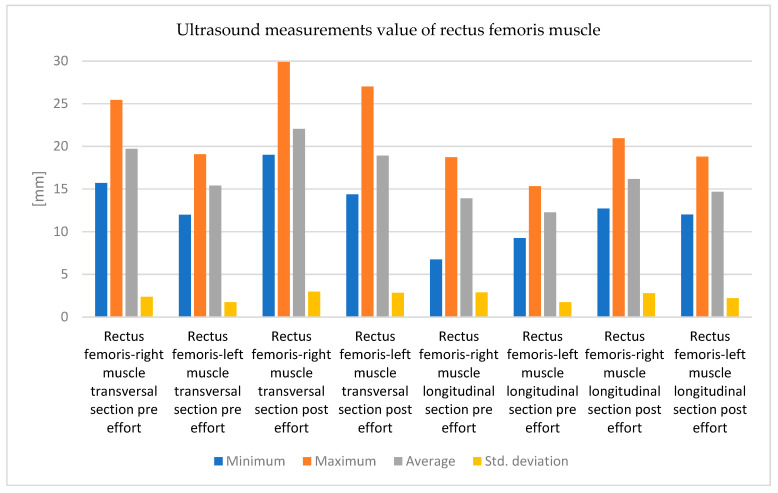
The variations in ultrasound measurements values (minimum, maximum, average, and standard deviation) of rectus femoris muscle (transversal and longitudinal measurement), pre effort and post effort.

It can be observed from the data presented in the table that there was an increase in the values of the echographic dimensions, approximately at the same level for both measured dimensions, which signifies the existence of an increase in muscle volume (Figure 7).

In the data presented in the table, we can observe an increase in the dimensions, more accentuated in the cross-sectional diameter, and compared to the evolution in the right lower limb, the increases are higher, which represents a response that may be based on the existence of a left/right asymmetry, this would require a more complete evaluation.

### 3.2. Results of Muscle Strength Assessment

The results of the parameters that characterize muscle strength are presented for each lower limb in terms of the values of maximum muscle strength, maximum average, and duration of maintenance of maximum load (maximum force), for the knee extensors (quadriceps). The results were analyzed from a descriptive as well as a statistical point of view.

#### Descriptive Analysis of the Values of Maximum and Mean Maximum Muscle Strength and Duration

*Right lower limb extension*

The values recorded for the right quadriceps are shown in Table 2, at T1 and T2 (6 months training interval).

**Figure 8:**
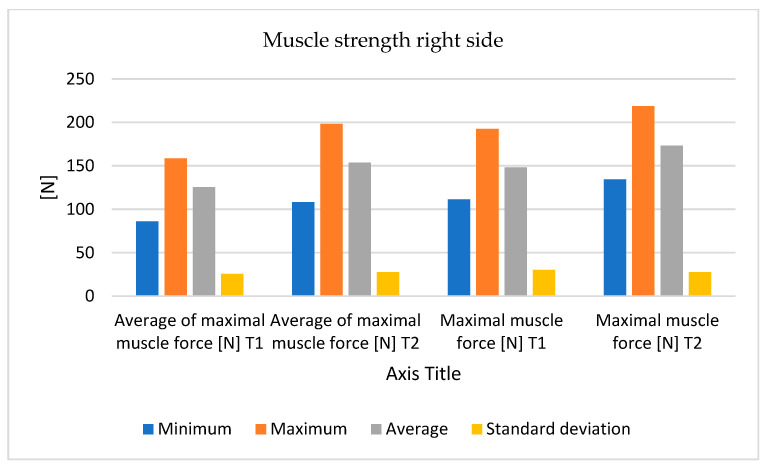
The variations in measurements values (minimum, maximum, average, and standard deviation) of muscle strength—right side.

**Figure 9:**
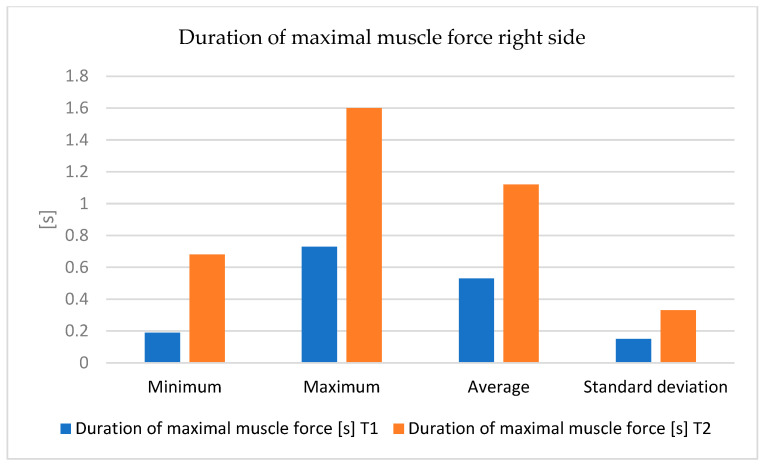
The variations in measurement values (minimum, maximum, average, and standard deviation) of the duration of maximal muscle strength—right side, between T1 and T2 moments.

An important progress was observed in the mean maximum strength and maximum force, but especially in the duration of maintenance of the maximum loading force (Figure 8 and Figure 9).

*Left lower limb extension*

The left quadriceps muscle strength values are shown in Table 3, two times that of T1 and T2.

**Figure 10:**
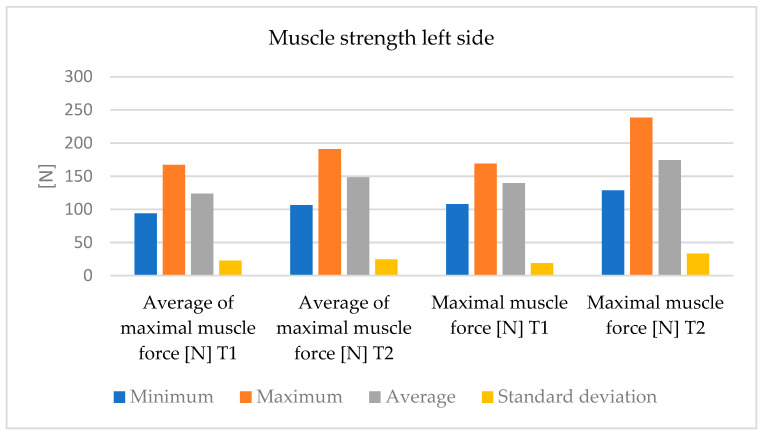
The variations in measurements values (minimum, maximum, average and standard deviation) of muscle strength—left side.

**Figure 11:**
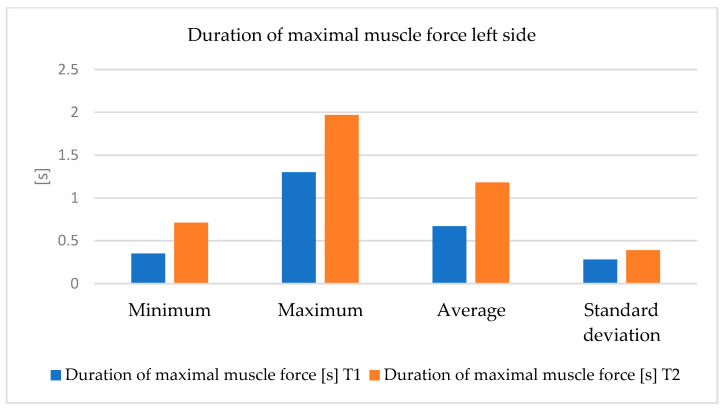
The variations in measurement values (minimum, maximum, average and, standard deviation) of the duration of maximal muscle strength—left side, between T1 and T2 moments.

We also observed significant progress in the duration of load maintenance. However, this progress is smaller than in the right lower limb (Figure 10 and Figure 11).

### 3.3. Results of Isoinertial Parameters

The values of the coefficient R1, in the Quadriceps (Qu) muscle group, before (time T1) and after (time T2) the training program is shown in Table 4.

**Figure 12:**
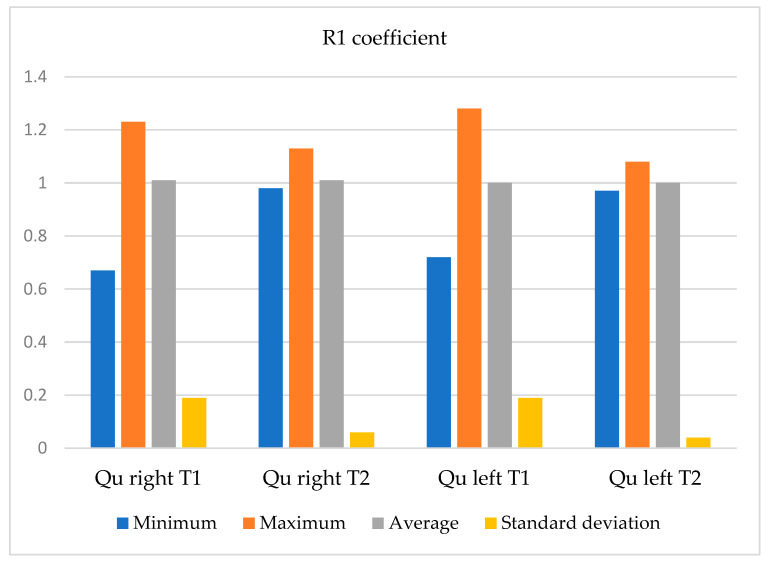
The variations in measurement values (minimum, maximum, average, and standard deviation) of the R1 coefficient, between T1 and T2 moments.

**Figure 13:**
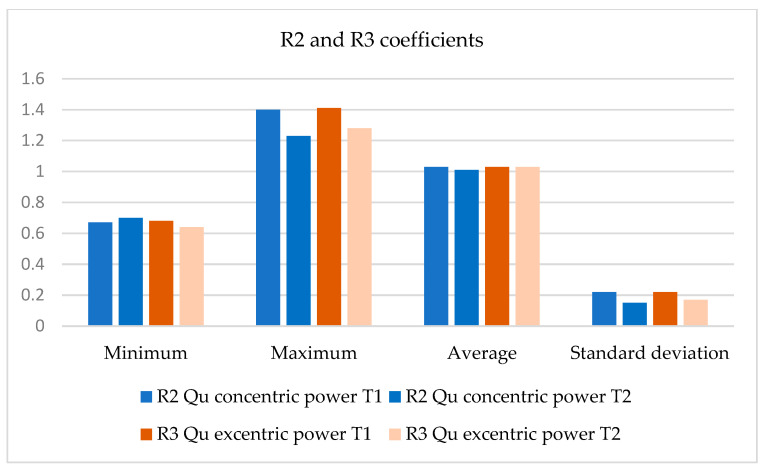
The variations in measurement values (minimum, maximum, average and, standard deviation) of the R2 and R3 coefficients, between T1 and T2 moments.

The symmetry coefficients R2 and R3 are presented for the quadriceps muscle group, before (T1) and after (T2) going through the training program, for the group of 11 sportswomen, are presented in Figure 12 and Figure 13.

### 3.4. Statistical Analysis of Measurement Results (Ultrasound, Muscle Strength Assessment and Isoinertial Parameters)

#### 3.4.1. Statistical Analysis of Ultrasound Measurement Results

In Table 5 and Table 6, the statistical indicators of the variation in the ultrasound signal values for the measurements of the transverse and longitudinal dimensions of the right and left rectus femoris muscle, pre and post exercise, are shown.

In the case of the right rectus femoris muscle the statistical analysis indicates a large magnitude of difference in the cross-sectional diameter between the two moments. The two-tailed *t*-test shows a statistically significant difference in means, *p* = 0.002, t_observed_ = −3.76 and df = 10, with large effect size.

Statistically, there is a large difference between the two assessment time points in longitudinal section size; the bilateral *t*-test shows a statistically significant difference in means, *p* = 0.0001, t_observed_ = −3.5 and df = 10, with large effect size.

At the level of the left rectus femoris, statistical indicators show a large difference in cross-sectional dimension values, and the two-tailed *t*-test shows a statistically significant difference in means, *p* = 0.0001, t_observed_ = −4.6 and df = 10, with large effect size.

Statistical analysis indicates a large difference in longitudinal section size values between the two assessments, and the two-tailed *t*-test shows a statistically significant difference in means, *p* = 0.0001, t_observed_ = −2.39 and df = 10, with a large effect size.

#### 3.4.2. Statistical Analysis of the Values of the Mean Maximum Extensor and Maximum Maximal Muscle Force Before and After the Training Program

Table 5 and Table 6 show the statistical indicators of the variation in the values of the mean maximum extension and maximum extension maximum muscle force.

The statistical analysis of the variation in the values of the maximum mean muscle strength in extension, indicates a large difference in magnitude between the two moments of the quadriceps force, which in the right lower limb, by bilateral *t*-test, shows a statistically significant difference in means, *p* = 0.0001, t_observed_ = −5.9 and df = 10, with large effect size. In the case of the left lower limb the statistical analysis indicates a very large magnitude of the difference between the two moments of the maximum mean strength. The two-tailed *t*-test shows a statistically significant difference in means, *p* = 0.006, t_observed_ = −3.48 and df = 10, with a large effect size.

For the maximum muscle strength in the lower limb (straight right lower limb) the statistical analysis indicates a large magnitude of difference between the two moments for the maximum quadriceps muscle force. The two-tailed *t*-test shows a statistically significant difference in means, *p* = 0.0001, t_observed_ = −4.99 and df = 10, with a large effect size. In the case of the left lower limb between the two moments, we observed a very large difference in magnitude of maximum muscle force. The two-tailed *t*-test shows a statistically significant difference in means, *p* = 0.005, t_observed_ = −3.55 and df = 10, with a large effect size.

#### 3.4.3. Statistical Analysis of Isoinertial Parameters

In Table 5 and Table 6, the statistical indicators of the variation in the values of the coefficients R1, R2, and R3, for the quadriceps group (left/right), concentric strength, eccentric strength, for the group of 11 sportswomen, are shown.

In terms of this coefficient, statistically, the differences are small between the two times of evaluation, and the two-tailed *t*-test shows a statistically insignificant difference in means, *p* = 0.058, t_observed_ = −0.563 and df = 10, with a small effect size for the right rectus femoris.

In the case of the left quadriceps muscle, the differences are also small between the two times of the assessment, and the two-tailed *t*-test shows a statistically insignificant difference in means, *p* = 0.76, t_observed_ = −0.3 and df = 10, with a small effect size.

The statistical analysis indicates a small magnitude of difference between the two moments of the R2 coefficient, and the two-tailed *t*-test shows a statistically insignificant difference in means, *p* = 0.083, t_observed_ = 0.215 and df = 10, with a small effect size.

The statistical analysis indicates a very small magnitude of difference between the two moments of the coefficient R3, and the two-tailed *t*-test shows a statistically insignificant difference in means, *p* = 0.95, t_observed_ = 0.056 and df = 10, with a small effect size.

## 4. Discussion

The results obtained are, from our point of view, relevant for emphasizing the role of such training in obtaining left/right, left/right functional symmetry, but also for demonstrating how the eccentric and concentric contraction force is influenced.

Thus, in the case of the R1 coefficient for each of the muscle groups analyzed, it shows us that this coefficient approaches the value of 1, in the case of the quadriceps muscle, and statistically, there is no significant improvement in this coefficient.

As for the coefficients R2 and R3, which reflect the left/right symmetry for concentric and eccentric power, respectively, the results reveal a tendency to left/right symmetrization for concentric power, more accentuated than for eccentric power.

From a statistical point of view, there was no statistically significant symmetrization at the level of the quadriceps muscle for concentric power, and no statistically significant symmetrization tendency for eccentric power; however, there was a favorable evolution in terms of values. Analyzing the mean values of the recorded data, it can be seen that in the case of concentric power there was a tendency towards symmetry (left/right) which shows that eccentric training has led to a development of concentric strength in conditions of muscular balance. The increase in concentric power values is explained by the accumulation of energy during eccentric contraction, which also allows the acquisition of symmetry.

Relating our results to data from the literature, the existence of a tendency towards left/right functional symmetrization, as well as the significant improvement in eccentric and concentric power, is explained by the fact that during eccentric contraction, myofibrils undergo an active stretch phenomenon, which leads to an accumulation of internal energy. Some of this energy is released when stretching has ceased, but an increased residual force remains. This physiological mechanism is present in skeletal muscle and underlies the maintenance of orthostatic position, shock absorption, and the accumulation of energy required for concentric contraction, an aspect revealed in our study and also mentioned by LaStayo et al. [39].

In addition, the fact that an improvement in muscle control and coordination was obtained, is an aspect that eccentric training improves muscle function through neural activation as mentioned by Paschalis et al. [40].

Cormie et al. [41] mentioned that eccentric training improves concentric strength, an element also revealed by our study.

Also, with respect to maximal strength, there are studies that emphasize an aspect also highlighted by us, namely that isoinertial training develops maximal strength, with a consequence on joint amplitude, especially in changes in direction [42].

The evolution of these parameters is explained by the development of coordination, the same aspect was also revealed by Van Hooren [43] who explained this coordination by the fact that the athlete is stimulated by this type of training to develop his or her capacity for rapid load transfer, based on a neuromuscular adaptation mechanism perfected through repetitions.

The same phenomenon of neuromuscular adaptation, which underlies muscle control and coordination, has also been mentioned by Hernández-Davó et al. [44].

Mike et al. [45] in their study, showed that 4-week isoinertial training led to increases in muscle strength, explosive strength, maximal, and average power.

The fact that the results of the program that we performed resulted in an increase in maximal power, concentric power, and muscle strength in the muscle groups studied is also demonstrated by Ali [46], who argued that a goal kicking performance in soccer (kicking) requires a concentric/excentric balance.

This increase in muscle strength is explained by Franchi et al. [47] by the fact that isoinertial training leads to an addition of sarcomeres in series, which generates an increase in muscle fascicle length.

Our initiative to apply such a program to junior female soccer players, part of a competing team, proves to be a means also supported by other authors such as Fiorilli [48]. He observed that it is beneficial to introduce such challenges as early as possible in juniors because it stimulates kinetic energy gain and enhances the athlete’s ability to adapt to real game conditions. The study by Bruseghini et al. reveals that by performing a comparative between High-Intensity Interval Training (HIT) and isoinertial training (IRT), a significant change in muscle strength occurs in IRT can be observed [49]. The results obtained by us can be corroborated by the results of Bollinger et al. [50], who showed that isoinertial training represents a novel form of training that leads to an increase in muscle strength, even at low loading, as evidenced by electromyographic (EMG) recordings, which we also obtained using D-disk loading as well as realizing a small loading. The comparable results are explained by the fact that there is an activation of the quadriceps motor units that is almost maximal in the isometric, concentric, and eccentric phases even with low relative inertial loads. From a morpho-functional point of view at low loading there is a detachment of myosin filaments, with the existence of a large number of transversely detached dots, which allow for the sustainment of the execution speed under conditions of maintaining the neural command. As the resistance increases, these points are activated because a force must be produced to overcome the flywheel inertia which limits the speed of execution. In other words, the formation of myosinic acto-myosin complexes is activated, which generate the muscle force that is also evidenced by EMG.

The effect of increasing the strength and muscular power of the quadriceps was also evidenced by Naczk et al. [38], who by training 3 times a week for 5 weeks, observed an increase in quadriceps muscle strength and power, as well as an increase in countermovement jump (CMJ), squat jump (SJ), maximal power output achieved during as well as electromyography of quadriceps, and muscle mass.

The same authors in 2024, studied inertial training vs. conventional strength training as contradictory; without being able to confirm the superiority of isoinertial training as a result of conducting a review in which both types of training were analyzed at the elbow and knee joints. The authors found, on the other hand, that the effect of isoinertial training on knee extensors is clearly superior to conventional training [38].

Analyzing the literature, we found studies claiming that isoinertial training causes morphological changes and neural adaptations that lead to an increase in muscle strength, an increase in muscle cross-sectional area, and musculotendinous stiffness. All these are based on improved motor unit recruitment, rate coding (firing frequency), synchronous motor unit activity, and neuromuscular inhibition [38]. Of course, small samples make it hard to verify these assumptions, increasing the risk of invalid conclusions. Small samples reduce the ability to detect true improvements in strength, hypertrophy, or injury prevention. Individual differences in training history, muscle fiber composition, and adaptation rates can skew results when only a few participants are studied. This variability makes findings less stable and harder to replicate.

Based on the ultrasound measurements, our results are sustained by the findings of Beato [31] which report that 5–8 weeks of flywheel training can significantly increase muscle volume and cross-sectional area, with continued gains over 10–11 weeks. The mechanism is based on enhanced satellite cell activation and protein synthesis and also structural remodeling of sarcomeres and connective tissue. Increased fascicle length and distal muscle cross-sectional area (CSA) are also ultrasound aspects that could be found and are linked with an increase in power and jump performance [30,51]. FWT is connected with a rise in inflammatory markers (IL-6, TNF-α, MCP-1) and circulating extracellular vesicles within 2 h, that involve muscle stress.

## 5. Conclusions

Eccentric training leads to changes in muscle structure and is considered useful for improving eccentric muscle strength; ultrasound evaluation could be a way to monitor the effects of isoinertial training.

Isoinertial training generates a post-acute increase in muscle volume demonstrated by effects on quadriceps muscle, and the effect could be long-term strength gains of muscle size based on an increase in cross-sectional diameter. Consistent ultrasound evaluations help identify abnormal adaptations, track muscle growth, and reveal potential risks for tendon or muscle injuries.

This evolution is associated with an important progression that was observed in the mean maximum force and maximum strength, but especially in the duration of maintenance of the maximum loading force.

By analyzing the mean values of the recorded data, it can be seen that in the case of concentric power there is a tendency towards symmetry (left/right) which highlights the fact that eccentric training has led to a development of concentric force under conditions of muscular balance. The increase in concentric power values is explained by the accumulation of energy during eccentric contraction, which also allows the acquisition of symmetry.

### Limitations of the Study

This study has several limitations that should be acknowledged. The relatively small sample size (n = 11) was constrained by strict inclusion criteria (elite female soccer players without recent injuries, consistent training participation) and the limited availability of specialized isoinertial equipment. However, this limitation is partially mitigated by several factors:

- The highly homogeneous study population with low inter-individual variability (CV = 11–21%);
- Large effect sizes (Cohen’s d: 0.80–1.48) that compensate for the smaller sample, yielding statistical power of 0.86–0.99 for significant findings;
- Consistent results across multiple measurement methods (ultrasonography, dynamometry, isoinertial parameters);
- Alignment with sample sizes reported in similar studies of novel training technologies.

Nevertheless, our findings should be interpreted as proof-of-concept evidence that warrants replication in larger, more diverse samples. Additional limitations include the relatively short intervention period (8 weeks), which may not capture long-term adaptations; individual variability in factors such as fitness level, nutrition, and genetics that may influence training responses; potential measurement errors inherent in ultrasound assessment and dynamometry; and the lack of a control group, which limits causal inference. The acute post-training measurements may reflect temporary changes rather than stable adaptations. Future studies with larger sample sizes, longer follow-up periods, control groups, and diverse populations are needed to confirm and extend these findings.

## Figures and Tables

**Table 1:** Ultrasound measurement values of rectus femoris muscle (transversal and longitudinal measurement).

Parameters	Rectus Femoris Muscle Transversal Section Pre Effort [mm]		Rectus Femoris Muscle Transversal Section Post Effort [mm]		Rectus Femoris Muscle Longitudinal Section Pre Effort [mm]		Rectus Femoris Muscle Longitudinal Section Post Effort [mm]	
	**right**	**left**	**right**	**left**	**right**	**left**	**right**	**left**
Minimum	15.70	11.97	18.99	14.36	6.73	9.23	12.70	12.01
Maximum	25.43	19.08	29.89	27.01	18.72	15.32	20.94	18.78
Average	19.70	15.40	22.039	18.90	13.899	12.27	16.172	14.66
Std. deviation	2.37	1.75	2.98	2.85	2.89	1.73	2.79	2.20
Cohen d-test	0.865	1.48			0.80	1.21		
Coefficient of variation	0.12	0.11	0.135	0.15	0.21	0.14	0.17	0.15

**Table 2:** Muscle strength right side.

Subject	Average of Maximal Muscle Force [N]		Maximal Muscle Force [N]		Duration of Maximal Muscle Force [s]	
	T1	T2	T1	T2	T1	T2
Minimum	85.97	108.20	111.31	134.35	0.19	0.68
Maximum	158.70	198.26	192.70	218.69	0.73	1.60
Average	125.51	153.62	148.17	173.31	0.53	1.12
Standard deviation	25.56	27.55	30.34	27.73	0.15	0.33
Cohen d-test	1.06		0.86		2.32	
Coefficient of variation [%]	20.37	17.93	20.47	16.00	29.15	29.45
Progress [%]	22.40		16.96		113.67	

**Table 3:** Muscle strength left side.

Subject	Average of Maximal Muscle Force [N]		Maximal Muscle Force [N]		Duration of Maximal Muscle Force [s]	
	T1	T2	T1	T2	T1	T2
Minimum	93.98	106.60	107.87	128.56	0.35	0.71
Maximum	167.20	190.74	169.16	238.30	1.30	1.97
Average	123.91	148.48	139.70	174.33	0.67	1.18
Standard deviation	22.84	24.55	18.91	33.47	0.28	0.39
Cohen d-test	1.04		1.27		1.52	
Coefficient of variation [%]	18.43	16.54	13.53	19.20	41.30	33.03
Progress [%]	19.83		24.79		76.84	

**Table 4:** Coefficient R1, R2 şi R3 (Qu—quadriceps).

	R1				R2		R3	
Subject	Qu Right		Qu Left		Qu Concentric Power		Qu Excentric Power	
	T1	T2	T1	T2	T1	T2	T1	T2
Minimum	0.67	0.98	0.72	0.97	0.67	0.70	0.68	0.64
Maximum	1.23	1.13	1.28	1.08	1.40	1.23	1.41	1.28
Average	1.01	1.01	1.00	1.00	1.03	1.01	1.03	1.03
Dev.stan Standard deviation	0.19	0.06	0.19	0.04	0.22	0.15	0.22	0.17
Cohen d’test	0.02		0.01		0.09		0.02	
Coeff of variation [%]	19.08	5.55	18.98	4.27	21.80	14.57	21.16	16.67
Progress [%]	−0.28		−0.15		−1.65		−0.33	

**Table 5:** Statistic indicators.

		STATISTIC INDICATORS for Difference (Post–Pre Effort)		*t*-Test			
Side	Values	Average	95% Confidence Interval	Effect Size (Cohen D’test)	t_observed_	df	*p* *
Right	Ultrasound transversal sections	−2.33	−3.65; −1.014	0.86	−3.76	10	0.002
	Ultrasound longitudinal sections	−2.27	−3.34; −1.2	0.8	−4.5	10	0.0001
Left	Ultrasound transversal sections	−3.5	−5.12; −1.87	1.48	−4.6	10	0.0001
	Ultrasound longitudinal sections	−2.39	−3.15; −1.63	1.21	−2.39	10	0.0001
Right	Average maximal muscle strength	−28.11	−38.72;−17.5	1.06	−5.9	10	0.0001
	Maximal muscle strength	−25.13	−36; −13	0.86	−4.99	10	0.001
Left	Average maximal muscle strength	−24.56	−40.3; −8.84	1.04	−3.48	10	0.006
	Maximal muscle strength	−34.63	−56.3; −12.95	1.27	−3.55	10	0.005
Right	R1	−0.034	−0.169; 0.101	0.02	−0.563	10	0.58
Left	R1	−0.019	−0.152; 0.115	0.01	−0.3	10	0.76
	R2	0.017	−0.159; 0.193	0.09	0.215	10	0.83
	R3	0.003	−0.135; 0.142	0.02	0.056	10	0.95

* *p* = 0.05.

**Table 6:** Average difference in measurements.

Side	Values	Difference (Post-Pre)	Coefficient of Variation [%]	Size of the Difference	Progress	Null Hypothesis
right	Ultrasound transversal sections	−2.33	13	High	Significant	Reject
	Ultrasound longitudinal sections	−2.27	17	High	Significant	Reject
left	Ultrasound transversal sections	−3.5	15	High	Significant	Reject
	Ultrasound longitudinal sections	−2.39	15	High	Significant	Reject
right	Average maximal muscle strength	−28.115	22.4	Great	Significant	Reject
	Maximal muscle strength	−25.13	16,96	Great	Significant	Reject
left	Average maximal muscle strength	−24.56	19.8	High	Significant	Reject
	Maximal muscle strength	−34.63	24.8	High	Significant	Reject
right	R1	−0.034	−0.28	Very small	No statistical significance	Not rejected
left	R1	−0.019	−0.15	Very small	No statistical significance	Not rejected
	R2	0.017	−1.65	Small	No statistical significance	Not rejected
	R3	0.003	−0.33	Very small	No statistical significance	Not rejected

## Data Availability

The original contributions presented in this study are included in the article. Further inquiries can be directed to the corresponding author.
